# Ultra-Smooth and Efficient NiO/Ag/NiO Transparent Electrodes for Flexible Organic Light-Emitting Devices

**DOI:** 10.3390/mi13091511

**Published:** 2022-09-12

**Authors:** Yu Bai, Yahui Chuai, Yingzhi Wang, Yang Wang

**Affiliations:** 1 School of Electronic Information Engineering, Changchun University of Science and Technology, Changchun 130022, China; 2 School of Physics, Changchun University of Science and Technology, Changchun 130022, China

**Keywords:** flexible OLEDs, NiO, transparent electrode

## Abstract

We demonstrate highly flexible and efficient organic light-emitting devices (OLEDs) using an ultra-smooth NAN anode. A template-stripping process was employed to create an ultra-smooth NAN anode on a photopolymer substrate. The flexible OLEDs obtained by this method maintained good electroluminescent properties and mechanical stability after bending. The efficiency of the flexible OLEDs was improved by 30.6% compared with conventional OLEDs deposited on PET/ITO substrate due to the enhanced hole injection from the ultra-smooth anode.

## 1. Introduction

In recent years, transparent conductive films have been used as electrodes in a variety of optoelectronic devices, such as liquid crystal display (LCD), organic light-emitting diodes (OLEDs), and solar cells. Earlier transparent conductive materials were oxides. Since the 1960s, indium tin oxide (ITO) has become the most representative transparent conductive material and is widely used [1,2,3,4]. It has a sheet resistance in the range of 20–500 Ω/□, and it boasts some significant advantages, such as high visible light transmittance and mature preparation technology [5]. However, it also has clear disadvantages; for instance, it is fairly “brittle” and, therefore, is not suitable for flexible OLEDs.

There are several alternatives that have been reported for ITO, such as carbon nanotubes [6,7,8,9], conducting polymers [10,11,12], graphene [13,14,15,16], metallic nanowires [17,18,19], and metallic grids [20,21,22]. However, many of these alternative materials suffer from either large sheet resistance [9,14] or high surface roughness [17,18,19,20], which cause low hole injection and reduced efficiency for OLEDs. Therefore, novel transparent electrodes with good optoelectrical properties and mechanical flexibility with low cost and low-temperature processing remain a big challenge.

Since the 1950s, research has shown that, if metal layers are placed into proper dielectric layers to form laminated constructions, the transmittance of the metal layers can be effectively enhanced [23,24]. Moreover, compared with the single-layer insertion of transparent conductive oxide films, laminated transparent conductive films yield a series of advantages, such as lower resistivity, near-room-temperature preparation technology, lower cost, good machinability, suppleness, and design flexibility. As such, research on dielectric−metal−dielectric (DMD) conductive film has gained traction in recent years [25]. However, for a usual flexible substrate, if DMD electric films are directly deposited on it, the high temperature of the deposition damages the substrate, and the deposited dielectric films are rough.

In this article, a nickel oxide (NiO)/silver (Ag)/nickel oxide (NiO) film with an ultra-smooth surface morphology is fabricated on a plastic substrate by employing a template-stripping technique and is used as an anode in flexible OLEDs. The NiO/Ag/NiO (NAN) OLEDs show superiority in both flexibility and mechanical stability. Moreover, the efficiency is 30.6% enhanced compared with conventional ITO OLEDs due to the enhanced hole injection at the ultra-smooth NAN–organic interface.

## 2. Materials and Methods

The NAN films were deposited on a precleaned, polished Si substrate by electron beam evaporation at room temperature. The evaporation rates of NiO and Ag were 0.09–0.11 and 0.9–1.1 nm/s, respectively, at pressures below 2.0 × 10^−3^ Pa. The hole-only devices and OLEDs were fabricated by thermal evaporation at room temperature. The evaporation rates of the organic material and Al were 0.1–0.2 and 0.9–1.1 nm/s, respectively, at pressures below 5.0 × 10^−4^ Pa. The active areas of the devices were all 1 × 1 mm^2^. The sheet resistance was measured using a four-probe method. An HMS-3000 Hall effect measurement system was used to measure the carrier concentration and Hall mobility of the NAN films, with an applied magnetic field of 0.55 T. A KP Technology Ambient Kelvin probe system package was used to measure the surface work function of the NAN films. A Shimadzu SPM-9700 was used to capture the AFM pictures. The J–V characteristics of the hole-only devices and OLEDs were measured using a computer-controlled Keithley 2611 source meter. The luminance of the OLEDs was measured using a Konica Minolta CS-100A luminance meter. The electroluminescent (EL) emission spectra were recorded using an Ocean Optics fiber spectrometer. The measurements were all conducted in air at room temperature.

## 3. Results and Discussion

**Figure 1:**
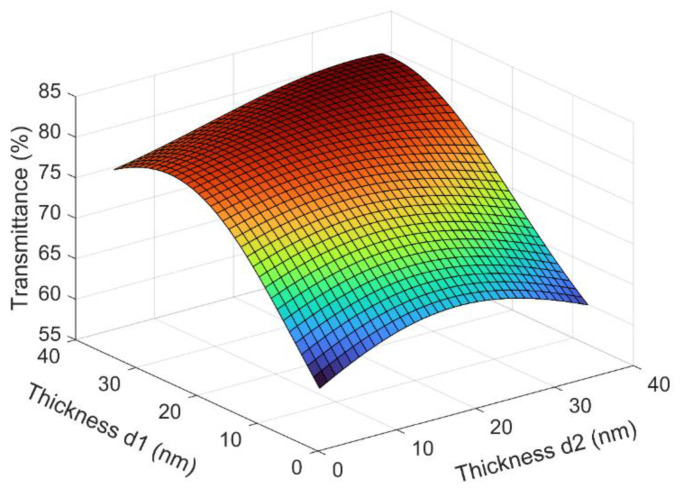
Calculated average transmittance of the polymer/NAN structures as a function of the thickness of the two NiO layers. The wavelength was between 380 nm to 780 nm. The thickness of Ag was fixed at 11 nm.

The transmittance in the visible range is an important parameter for transparent conductive films as anodes for OLEDs. If the Ag layer in NAN films is too thin, its conductivity is compromised, and if it is too thick, the transmittance is not good. Previous experiments have pointed to an Ag thickness of 11 nm as the best balance between conductivity and transmittance. After determining the thickness of the Ag, we performed optical simulations for different thicknesses of the NiO layers as they were combined with the Ag, as shown in Figure 1. We can see that, when both NiO layers were at 35 nm of thickness, the NAN film had a high average transmittance in the wavelength of 380 nm–780 nm. Therefore, we adopted a NiO (35 nm)/Ag (11 nm)/NiO (35 nm) structure for the NAN thin film in this article, and its transmittance was above 80% in the green band.

**Figure 2:**
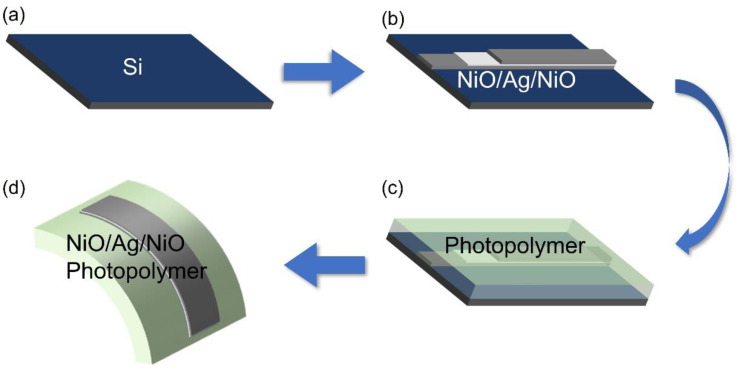
Steps of fabricating ultra-smooth NAN film by template stripping. (**a**) Si substrate was cleaned as the template. (**b**) NAN film was deposited onto the Si template by electron beam evaporation. (**c**) Photopolymer (NOA63) was spin-coated as the backing film. (**d**) The NAN film adhered to the cured photopolymer film was stripped.

We adopted a stripping technique to fabricate the ultra-smooth NAN thin film, as shown in Figure 2. First, a cleaned silicon (Si) template was loaded into an electron beam evaporation chamber. An 81 nm thick NAN film was grown on the Si template. Then, a 1 mm thick photopolymer (NOA63, Norland) was spin-coated on the NAN film at 1000 rpm for 20 s. After that, the photoresist was solidified with ultraviolet light under a 100 w light source for 8 min. Lastly, the cured photoresist was stripped off the silicon wafer. Because the adhesion between NiO and Ag, as well as between NiO and the photoresist, was stronger than that between NiO and Si, the NAN film could be stripped off the silicon wafer. With this stripping technique, an ultra-smooth NAN flexible substrate was obtained, and the process could be repeated decently in a large area.

**Figure 3:**
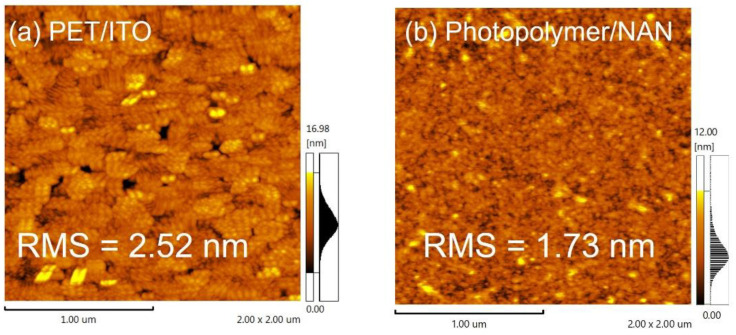
AFM images of (**a**) PET/ITO and (**b**) Photopolymer/NAN.

PET/ITO is a currently popular flexible transparent anode. We, therefore, compared the surface roughness (RMS) of the ultra-smooth Photopolymer/NAN with PET/ITO. Figure 3 shows AFM images of the surface roughness of Photopolymer/NAN and PET/ITO. It was observed that, in the scanned area of 2 μm × 2 μm, the average surface roughness (RMS) values of PET/ITO and Photopolymer/NAN were 2.52 nm and 1.73 nm, respectively. The average roughness (Ra) values of PET/ITO and Photopolymer/NAN were 2.06 nm and 1.26 nm, respectively. Because ITO film is grown directly on a PET surface, it is riddled with studs and, therefore, is very rough, whereas on NAN films, studs only pop up occasionally and sparingly and are very scattered, rather than concentrated on the film surface. Therefore, in general, the NAN film could be categorized as an ultra-smooth film. We also measured the work function, carrier density, and Hall mobility of the NAN film. The work function was 5.3 eV, the carrier density was 8.559 × 10^21^ cm^−3^, and the Hall mobility was 14.23 cm^2^/Vs. A calculated sheet resistance of 7.6 Ω/□ was achieved, which was lower than those of most commercial ITO electrodes [26].

The data above show that the carrier density and carrier mobility of the NAN film were like those of ITO, yet its work function (5.3 eV) was significantly higher than that of ITO (4.7 eV). Take the most common hole transport material, N,N′-diphenyl-N,N″-bis(1,1′-biphenyl)-4,4′-diamine (NPB), as an example: its HOMO level is 5.4 eV. That means, if we used NAN film as an anode, because its work function was nearly at the HOMO level of NPB, it would provide an edge for the hole to be injected into the anode. Furthermore, because the hole now needed to jump a smaller energy level difference, there was no need to add a hole injection layer between the anode and the hole transport layer. This would greatly reduce the fabrication complexity of OLED devices.

**Figure 4:**
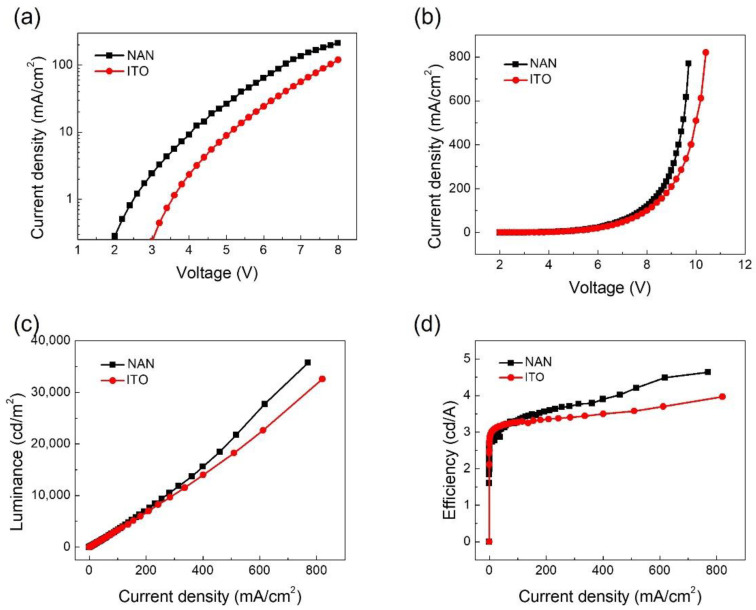
Performance of OLEDs with NAN and ITO anodes. (**a**) Current density–voltage characteristics of hole-only devices. (**b**) Current density–voltage characteristics, (**c**) luminance–current density characteristics, and (**d**) efficiency–current density characteristics.

To demonstrate the hole injection capacity of the NAN ultra-smooth film, we fabricated two types of hole-only devices. Their structures were NAN/NPB (60 nm)/Al (100 nm) and ITO/NPB (60 nm)/Al (100 nm). In the two devices, the roles of the NPB were hole injection and transport layer. Because the work function of Al was 4.2 eV and the LUMO level of NPB was 2.4 eV, a huge transition barrier was created for the electrons so that it was very difficult for them to be injected from the cathode. Figure 4a shows the voltage–current density curve of the hole-only device. The current density of the NAN film anode hole-only device was greater than that of the ITO anode hole-only device. This shows that the ultra-smooth NAN film was very conducive to enhancing the hole injection efficiency.

We then used the ultra-smooth NAN film and the PET/ITO as anodes and fabricated two types of OLEDs. Tris-(8-hydroxyquinoline) aluminum (Alq3) was used as an emitting layer. The device structures were NAN/NPB (60 nm)/Alq3 (70 nm)/LiF (1 nm)/Al (100 nm) and ITO/NPB (60 nm)/Alq3 (70 nm)/LiF (1 nm)/Al (100 nm). Figure 3b–d compares the performance curves of the NAN-anode-based and ITO-anode-based OLEDs. The maximum efficiency of the NAN-anode-based OLED was 4.7 cd/A, whereas that of the ITO-anode-based OLED was 3.6 cd/A; that was a 30.6% enhancement in efficiency. Such an enhancement came from the greater work function and the smaller surface roughness of the NAN film, both contributing to a higher hole injection efficiency.

**Figure 5:**
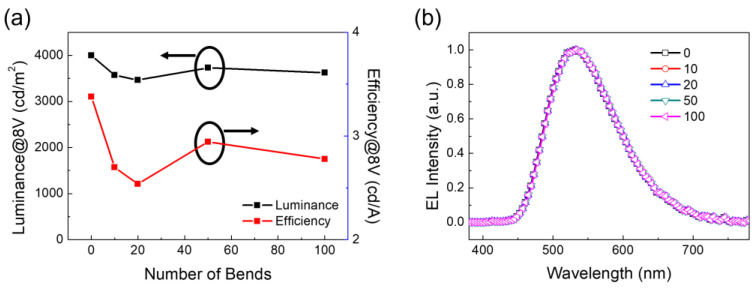
Mechanical stability of the flexible OLEDs. (**a**) Luminance and efficiency at 8 V as a function of the number of bending cycles. (**b**) Comparison of EL spectra before and after repeated bending.

A bending test was conducted on the NAN-anode-based OLED. We bent it into a U shape, almost folding it. Figure 5 shows the luminosity, efficiency, and luminescence spectrum of the device after repeated bending and at a voltage of 8 V. It can be observed that, after 0–100 times of bending, the luminosity and efficiency of the device did not change much. Moreover, the luminescence spectrum did not move much, demonstrating the good mechanical stability of this ultra-smooth NAN film.

## 4. Conclusions

In conclusion, an ultra-smooth flexible NAN film was prepared using a stripping technique. It was then used as an anode in the fabrication of a flexible OLED. Because the NAN had a higher work function and smoother surface compared to ITO, it enhanced the hole injection efficiency and, therefore, the luminosity and efficiency of the OLED. Furthermore, bending tests demonstrated the strong mechanical stability of the NAN anode made with the stripping technique. This research showed that films prepared using such stripping and lamination methods could be good candidates for anodes in indium-free, flexible OLEDs in a large-scale fabrication context.

## Data Availability

Not applicable.
